# Optimizing anesthesia and delivery approaches for dosing into lungs of mice

**DOI:** 10.1152/ajplung.00046.2023

**Published:** 2023-07-04

**Authors:** Yurim Seo, Longhui Qiu, Mélia Magnen, Catharina Conrad, S. Farshid Moussavi-Harami, Mark R. Looney, Simon J. Cleary

**Affiliations:** Department of Medicine, University of California, San Francisco, California, United States

**Keywords:** dosing, intranasal, intratracheal, lung, mouse

## Abstract

Microbes, toxins, therapeutics, and cells are often instilled into lungs of mice to model diseases and test experimental interventions. Consistent pulmonary delivery is critical for experimental power and reproducibility, but we observed variation in outcomes between handlers using different anesthetic approaches for intranasal dosing in mice. We therefore used a radiotracer to quantify lung delivery after intranasal dosing under inhalational (isoflurane) versus injectable (ketamine/xylazine) anesthesia in C57BL/6 mice. We found that ketamine/xylazine anesthesia resulted in delivery of a greater proportion (52 ± 9%) of an intranasal dose to lungs relative to isoflurane anesthesia (30 ± 15%). This difference in pulmonary dose delivery altered key outcomes in models of viral and bacterial pneumonia, with mice anesthetized with ketamine/xylazine for intranasal infection with influenza A virus or *Pseudomonas aeruginosa* developing more robust lung inflammation responses relative to control animals randomized to isoflurane anesthesia. Pulmonary dosing efficiency through oropharyngeal aspiration was not affected by anesthetic method and resulted in delivery of 63 ± 8% of dose to lungs, and a nonsurgical intratracheal dosing approach further increased lung delivery to 92 ± 6% of dose. The use of either of these more precise dosing methods yielded greater experimental power in the bacterial pneumonia model relative to intranasal infection. Both anesthetic approach and dosing route can impact pulmonary dosing efficiency. These factors affect experimental power and so should be considered when planning and reporting studies involving delivery of fluids to lungs of mice.

**NEW & NOTEWORTHY** Many lung research studies involve dosing fluids into lungs of mice. In this study, the authors measure lung deposition using intranasal (i.n.), oropharyngeal aspiration (o.a.), and intratracheal (i.t.) dosing methods in mice. Anesthetic approach and administration route were found to affect pulmonary dosing efficiency. The authors demonstrate that refinements to dosing techniques can enable reductions in the number of animals needed for bacterial and viral pneumonia studies.

## Graphical abstract

**Figure:**
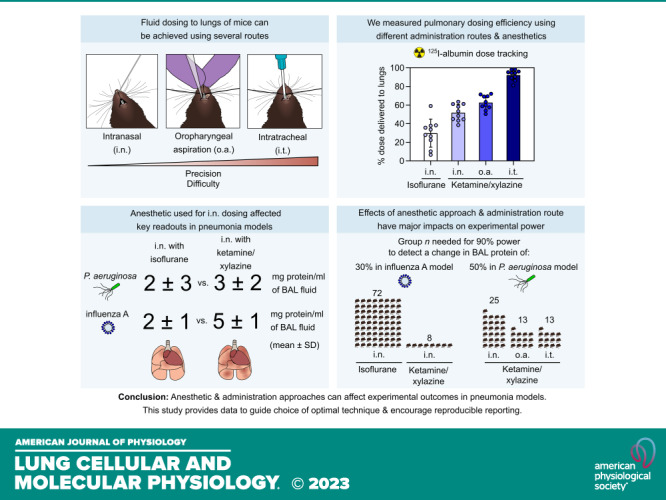

## INTRODUCTION

Studies investigating lung infections, lung injury, allergic airway inflammation, lung fibrosis, lung cancer, and lung stem cell biology often require delivery of experimental agents to lungs of mice. Administration routes for bolus dosing of fluids into lungs include intranasal (i.n.) dosing, intratracheal (i.t.) dosing, and dosing through oropharyngeal aspiration (o.a.). Choice of dosing route is an important decision in study design as experimental outcomes can be altered by the quantity of dose delivered to lungs or to extrapulmonary tissues. Different dosing routes also vary in anesthetic requirements, invasiveness, and technical difficulty.

To guide experimental approach, a previous study assessed the effect of various factors, including type of anesthetic, on the distribution of intranasal doses into BALB/c mice. This study concluded that either injectable (Avertin) or inhaled (isoflurane and halothane) anesthetics resulted in similar delivery to lungs (1). Since this influential report, several factors have changed. Safety concerns have led to a decline in the use of both Avertin and halothane (2, 3). Increased availability of knockouts and transgenics on the C57BL/6 (B6) background has led to B6 mice becoming the most widely used laboratory strain. In addition, minimally invasive approaches for dosing via oropharyngeal aspiration and intratracheal routes have been developed, which can more efficiently deliver fluids to lungs relative to intranasal dosing (4–6).

In our previous work, we noticed that intranasal doses passed more readily into the nostrils in studies where B6 background mice were anesthetized with ketamine/xylazine compared with experiments in which mice were anesthetized with isoflurane (7, 8), but we did not know whether anesthetic used during dosing was affecting pulmonary deposition. To guide future studies using these mice and anesthetics, we therefore measured the effect of anesthetic approach on intranasal delivery of fluid to lungs of B6 mice. As we have used and refined oropharyngeal aspiration and intratracheal methods, we also measured dose distribution using these administration routes.

We found striking effects of both anesthetic approach and dosing route on the efficiency of pulmonary delivery. Our results will be useful to guide the design of experiments with improved reproducibility—an international biomedical research policy goal (9, 10). In addition, our findings indicate potential strategies to reduce the number of mice needed to produce clear results from experiments involving dosing of fluids into lungs.

## METHODS

### Animals

C57BL/6 background mice (Jax No. 000664) were housed at the University of California, San Francisco (UCSF) Parnassus Laboratory Animal Resource Center’s specific pathogen-free facility. Male and female mice were used in equal numbers per group at ages 6–14 wk. Mice were kept on a 12-h light-dark cycle. Protocols were approved by the UCSF Institutional Animal Care and Usage Committee.

### Anesthesia

Mice were anesthetized either by inhalation of isoflurane (4% in oxygen) or by intraperitoneal (i.p.) injection with ketamine (70 mg/kg) and xylazine (15 mg/kg) in normal saline. For fluid dosing to lungs, it is important not to overdose ketamine/xylazine, as higher doses can cause asphyxiation from aspirated fluid. For terminal anesthesia before collection of lung samples, mice were euthanized with ketamine (100 mg/kg) and xylazine (40 mg/kg) before exsanguination.

### Intranasal Dosing

Gel-loading pipette tips (Sorenson No. 13810) were used to introduce 50 µL of dose dropwise into the posterior opening of one nare. Mice were held upright for 20 s after dosing to allow aspiration of dose.

### Tracking Radiolabeled Albumin Doses

For quantitative and visual tracking of inoculum, we used 50 µL of phosphate-buffered saline (PBS) containing ^125^I-albumin (0.25 mg/mL, ∼2.5 KBq/mL, Jeanatope, Iso-Tex Diagnostics, Inc.) and Evans blue dye (1 mg/mL). Organ samples were collected 10 min after mice were dosed. Dose distribution was measured using a gamma counter (Packard 5000 series) against three standards containing 100% of injected dose.

### Influenza A Virus Infection Model

Mice were infected by the intranasal route with 50 plaque-forming units (p.f.u.) of influenza A virus (A/PR/8/34 H1N1) propagated with Madin–Darby canine kidney (MDCK) cells. For propagation, MDCK cells cultured in minimal essential medium (MEM) supplemented with 10% FBS and penicillin/streptomycin in a humidified incubator at 37°C and 5% CO_2_ were infected and cultured for 72 h. The supernatant containing the virus was then collected and stored at −80°C. Infectious virus was quantified by culturing dilutions of the viral stock with MDCK cells in a six-well plate for 1 h, followed by addition of an overlay of 1.2% Avicel RC-581 in MEM, culture for 72 h, formalin fixation, and staining with crystal violet for p.f.u. determination (11). Viral stocks were diluted in sterile PBS at 4°C before inoculation. Mice were dosed at zeitgeber time (ZT) 3–5 and handled under biosafety level 2 conditions.

### Bronchoalveolar Lavage Analysis

After terminal anesthesia and exsanguination, lungs were collapsed by opening the diaphragm and tracheal insertion of 20G stub needles. A 1 mL syringe containing 1 mL of phosphate-buffered saline was then washed in and out of the lungs three times to recover bronchoalveolar lavage (BAL) fluid. BAL cells were counted using a LUNA-II automated cell counter (Logos Biosystems), and BAL supernatant total protein was measured using a Pierce total protein assay (Thermo Scientific, No. 23225).

### Dosing by Oropharyngeal Aspiration

As previously described, anesthetized mice were placed on an intubation platform suspended by their upper incisors with the tongue gently pulled out of the mouth (5, 12). The fluid dose was then pipetted directly onto the distal oropharynx at 50 µL volume with both nares covered to obligate breathing through the mouth. After ∼30 s, mice were removed from the platform and placed supine until sample collection.

### Nonsurgical Intratracheal Dosing

Customized intubation and injection apparatus were prepared from a blunted 22G 1′′ Safelet IV Catheter (Nipro, No. CI+2225-2 C) customized into an endotracheal tube, and a blunted 28G insulin syringe (BD No. 329461) attached to PE-10 tubing (Fig. 4*A*). Cushions were added using cyanoacrylate glue and PU-40 or PE-20 tubing. Mice were positioned as with oropharyngeal aspiration dosing, with transillumination and adjustment of body position used for visualization of the larynx (Fig. 4*B*). Mice were then orotracheally intubated with correct placement confirmed by attaching a manometer to the endotracheal tube and checking for oscillation of water column with breathing movements (Fig. 4*C*) (13). The tubing attached to the syringe was then inserted into the catheter (as shown in Fig. 4*D*) for injection of dose at 50 µL volume followed by 120 µL air.

### *Pseudomonas aeruginosa* Infection Model

*Pseudomonas aeruginosa* (PAO1, ATCC No. BAA-47) was grown to a logarithmic phase in suspension in tryptic soy broth (TSB). Pellets of PAO1 were then resuspended in PBS at 4°C and adjusted to a density of 1 × 10^6^ colony-forming units (c.f.u.) per 50 µL inoculation volume for mouse infections.

Infected mice were given a subcutaneous dose of 0.5 mL of normal saline at 4 h after infection as fluid support. BAL fluid was collected as described earlier at 24 h after infection.

### Experimental Design and Statistical Analysis

Mice were randomly assigned to groups with blocking by cage, and samples were collected and quantified with investigators blinded to groups. For infection studies, the handler dosing mice was also blinded during dosing, with a second unblinded handler in control of anesthesia. Group *n* was set before study initiation and analysis and reflects numbers of individual subjects (mice). Where necessary, data were transformed before statistical testing according to distribution. Statistical analyses used InVivoStat 4.4 (body weight and power analysis) or GraphPad Prism 9 (other comparisons). The tests used for each analysis are stated in figure legends with *P* = 0.05 as α threshold. Data are reported as means ± standard deviation unless otherwise stated.

## RESULTS

In previous experiments, we noticed that B6 mice anesthetized with ketamine/xylazine smoothly aspirated intranasal doses, whereas intranasal doses sometimes bubbled back out of the nares of isoflurane-anesthetized mice (7, 8). As previous studies assessing effects of anesthesia on intranasal delivery to lungs used anesthetics or mouse strains not used in our protocols (1, 6, 14), we aimed to determine whether use of isoflurane or ketamine/xylazine anesthesia during intranasal dosing affected the delivery of dose to lungs of B6 mice.

**Figure 1.**
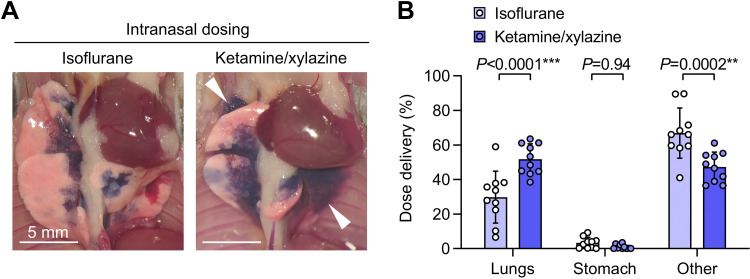
Increased pulmonary dosing efficiency with ketamine/xylazine vs. isoflurane anesthesia during intranasal dosing. *A*: B6 mice were given intranasal (i.n.) doses containing ^125^I-albumin radiotracer and Evans blue dye under either isoflurane or ketamine/xylazine anesthesia. Photographs show lungs after euthanasia and thoracotomy, with delivery of dye to more distal regions of the lungs with ketamine/xylazine anesthesia (white arrowhead). *B*: effect of anesthetic type on dose distribution quantified using radiotracer. Means ± standard deviation, *n* = 10. *P* values are from a repeated-measures two-way ANOVA with Holm–Šídák tests for effect of dosing route within each location.

We found that relative to isoflurane anesthesia, use of ketamine/xylazine anesthesia during intranasal dosing resulted in delivery of dose to more distal regions of lung (Fig. 1*A*) and increased pulmonary dosing efficiency (Fig. 1*B*). The proportion of dose not delivered to the lungs was not immediately swallowed, but either remained in the upper respiratory tract or was refluxed out of the nostrils (quantified as “Other” in Fig. 1*B*).

The intranasal dosing route is in widespread use in respiratory virus infection models. Current protocols suggest that handlers can use either isoflurane or ketamine/xylazine anesthesia during intranasal infection with influenza A virus (15). We therefore formally tested whether the effect of anesthetic approach on intranasal dosing to lungs could be a factor altering outcomes and reproducibility of studies of respiratory viral infection.

With one handler delivering anesthesia and a second handler blinded to anesthetic approach dosing and assessing mice, we randomized B6 mice to receive isoflurane or ketamine/xylazine anesthesia before intranasal doses containing 50 p.f.u. of PR8 influenza A virus.

**Figure 2.**
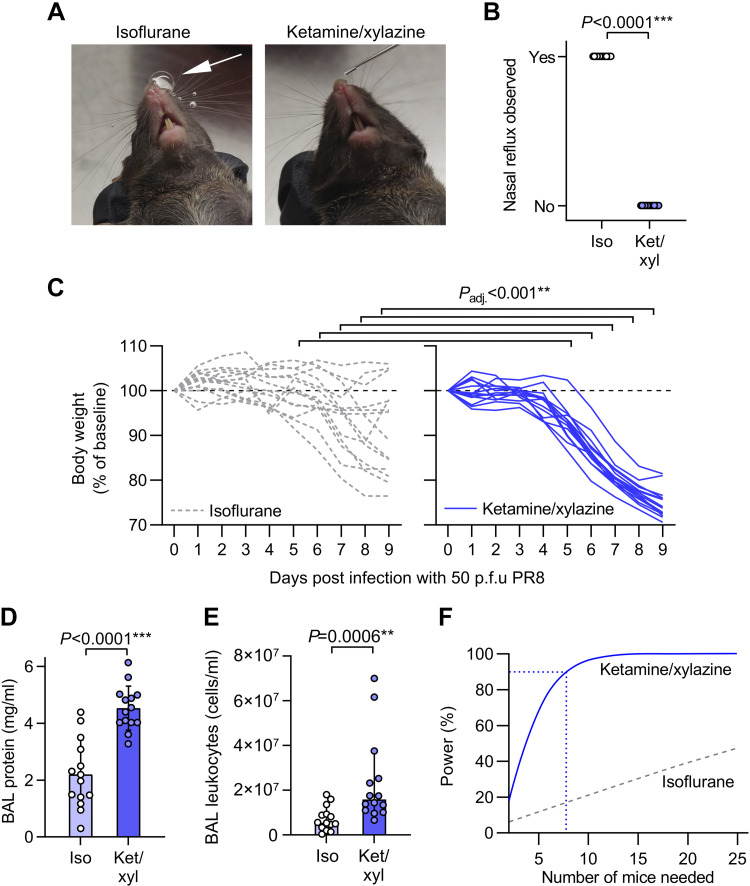
Increased body weight loss and lung inflammation after intranasal infection with influenza A virus under ketamine/xylazine relative to isoflurane anesthesia. *A*: mice were randomized to receive either isoflurane or ketamine/xylazine anesthesia for intranasal dosing with 50 plaque-forming units (p.f.u.) of H1N1 influenza A virus A/PR/8/1934 (PR8). Photographs of mice show presence of nasal reflux during intranasal dosing under isoflurane anesthesia (white arrow) but not ketamine/xylazine anesthesia. *B*: quantification of incidence of nasal reflux during intranasal dosing under isoflurane (iso) vs. ketamine/xylazine (ket/xyl). *C*: body weight changes over 9 days post infection. *D*: bronchoalveolar lavage (BAL) supernatant protein concentration at *day 9* postinfection. *E*: leukocyte counts from BAL fluid at *day 9* postinfection. *F*: output of power analysis using total protein data in *D* to estimate the number of mice needed per group to detect a 30% change in BAL protein concentration using an unpaired two-tailed *t* test. Means ± standard deviation except for *E* which shows medians ± 95% confidence intervals and was log_10_-transformed before analysis, *n* = 14. *P* values are from Fisher’s exact test (*B*), repeated-measures mixed-model approach with baseline values as covariates and adjusted (adj.) *P* values from Holm’s tests for effect of anesthesia within each time point (*C*), and unpaired two-tailed *t* tests (*D* and *E*).

We observed bubbling of dose back out of nares and down the philtrum in mice in our biodistribution study. During infection with PR8, the handler blinded to anesthesia approach therefore recorded whether dose reflux was observed. We found that isoflurane-anesthetized mice consistently refluxed some dose back out of their nares, whereas mice anesthetized with ketamine/xylazine smoothly aspirated doses without visible reflux (Fig. 2, *A* and *B*).

We also monitored body weight daily as an index of general health status. All mice anesthetized with ketamine/xylazine at time of infection had lost weight at *day 9*, but weight loss was significantly lower in the isoflurane-anesthetized group from 5 to 9 days postinfection, with some mice in the isoflurane group gaining weight after inoculation (Fig. 2*C*).

At 9 days postinfection, we collected bronchoalveolar lavage (BAL) fluid from infected mice to measure vascular leak and leukocyte recruitment into lung airspaces as indices of lung inflammation. Both supernatant protein concentration and leukocyte counts were higher in BAL fluid from ketamine/xylazine-anesthetized mice compared with isoflurane-anesthetized mice (Fig. 2, *D* and *E*).

Using the BAL protein data in Fig. 2*D*, we ran a power analysis to determine the group size needed for future experiments aimed at detection of a 30% change in BAL fluid protein concentration using unpaired two-tailed *t* tests. We found that nine mice per group would be needed to run such an experiment with 95% power with ketamine/xylazine anesthesia (Fig. 2*F*). In comparison, the isoflurane anesthesia approach would likely not be feasible for experimental use, as an experiment with 25 mice per group would still have less than 50% power (Fig. 2*F*).

Together, these results indicate that isoflurane anesthesia spares a reflex involving sensing of fluid in the upper airways and limitation of pulmonary aspiration. In contrast, use of ketamine/xylazine anesthesia circumvents this reflex, facilitating aspiration of a greater proportion of intranasal dose. This effect means that the two anesthesia approaches yield different efficiency and distribution of intranasal dosing efficiency to the lungs, affecting key outcomes in a respiratory virus infection model.

Compared with intranasal dosing, the oropharyngeal aspiration route involving aspiration from the distal oropharynx can result in less exposure of nasal sinuses to inoculum and increased dosing efficiency to the lungs. Since anesthetic type affected intranasal dosing, we sought to also determine whether different anesthesia approaches altered delivery of oropharyngeal aspiration doses to the lungs.

**Figure 3.**
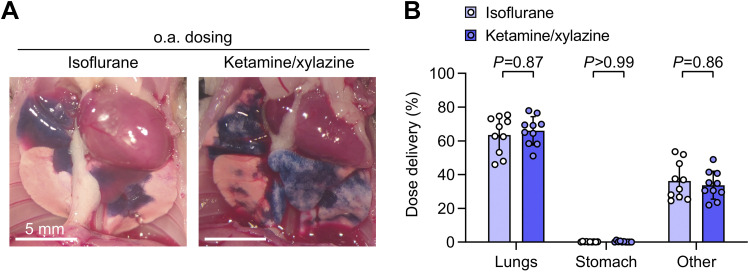
Pulmonary dosing efficiency using oropharyngeal aspiration is not altered by anesthetic type. *A*: B6 mice were given oropharyngeal aspiration (o.a.) doses containing ^125^I-albumin radiotracer and Evans blue dye under either isoflurane or ketamine/xylazine anesthesia and euthanized 10 min later. Assessment of lungs after thoracotomy showed bilateral delivery of dye to distal regions of lungs with both anesthetic types. *B*: no effect of anesthesia approach was found on biodistribution of radiotracer. Means ± standard deviation, *n* = 10. *P* values are from a repeated-measures two-way ANOVA with Holm–Šídák tests for effect of dosing route within each location.

Breath-holding responses were observed in some isoflurane-anesthetized mice after doses were dropped onto the oropharynx, but isoflurane-anesthetized mice eventually aspirated doses with nasal reflux and swallowing prevented by covering the nares and retracting the tongue. Breath holding was not observed in ketamine/xylazine-anesthetized mice given oropharyngeal aspiration doses, potentially resulting in more rapid aspiration over multiple breaths and patchier deposition (Fig. 3*A*). Tracking dose delivery quantitatively, we did not detect any effect of anesthesia approach on oropharyngeal aspiration dose deposition in the lungs (Fig. 3*B*).

We conclude, from this study, that anesthetic approach is therefore unlikely to have a major impact on dosing to the lungs via the oropharyngeal aspiration route.

**Figure 4.**
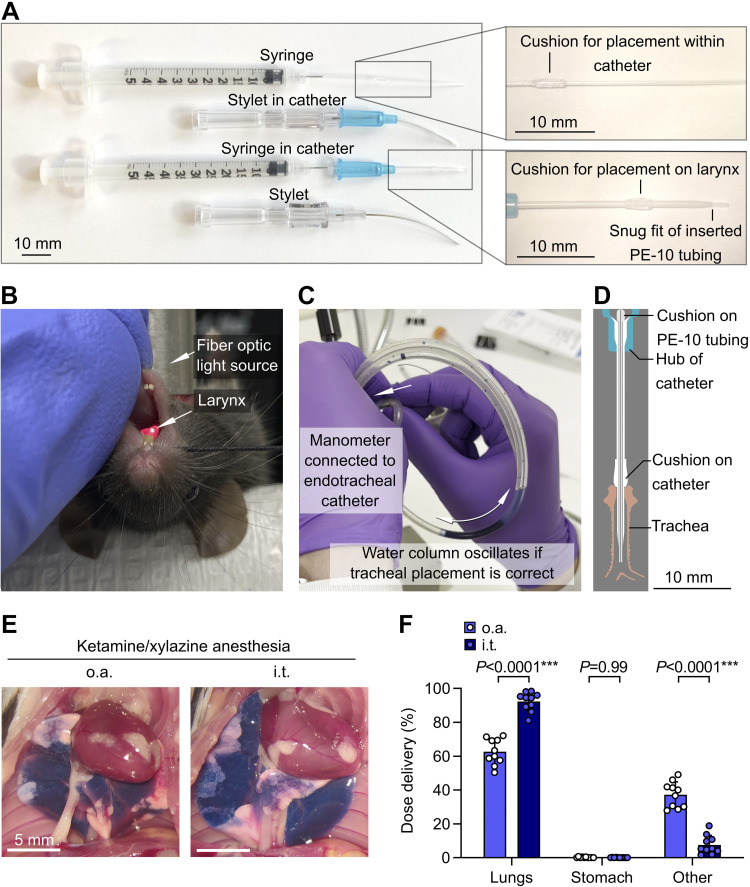
Increased pulmonary dosing efficiency with nonsurgical intratracheal dosing relative to oropharyngeal aspiration. *A*: customized catheters and syringes for orotracheal intubation and precise intratracheal injections through endotracheal tube. *B*: mouse on intubation platform showing direct visualization of laryngeal inlet through transillumination of the trachea. *C*: manometer used for confirmation of correct airway placement after orotracheal intubation. *D*: drawing showing cross section of tubing, catheter, and trachea. *E*: B6 mice were anesthetized with ketamine/xylazine and then given ^125^I-albumin and Evans blue by either oropharyngeal aspiration (o.a.) or intratracheal (i.t.) routes. Photographs show representative dose distribution. *F*: effect of administration approach on dose distribution quantified using radiotracer. Means ± standard deviation, *n* = 10. *P* values are from a repeated-measures two-way ANOVA with Holm–Šídák tests for effect of dosing route within each location.

Nonsurgical intratracheal dosing approaches have potential for more precise lung dosing relative to intranasal and oropharyngeal aspiration dosing. Previous studies suggest that oropharyngeal aspiration dosing can yield similar dosing efficiency compared with intratracheal dosing, but these reports have not directly measured lung delivery using the latest nonsurgical intratracheal approaches (16–19). We have optimized an approach for intratracheal dosing involving direct visualization of the larynx, orotracheal intubation with customized catheter, confirmation of airway placement using a manometer, and then injection using a customized syringe (5, 6, 12, 13) (Fig. 4, *A*–*D*). We therefore sought to measure pulmonary dosing efficiency using our nonsurgical approach for intratracheal dosing, comparing to a control group dosed with the oropharyngeal aspiration approach, using ketamine/xylazine anesthesia.

We found that intratracheal dosing yielded increased pulmonary dose deposition relative to oropharyngeal aspiration dosing (Fig. 4, *E* and *F*). This result is indicative that although oropharyngeal aspiration and intratracheal routes deliver the majority of injected dose to the lungs, intratracheal dosing might be desirable in situations where precise dosing to lungs is needed.

We have previously modeled bacterial lung infections by infecting mice using the intranasal, oropharyngeal aspiration, and intratracheal routes (20–23). To guide future studies using bacterial pneumonia models and directly compare performance of each of the dosing methods used in our work, we studied the effects of administration approach on lung inflammation following infection with *Pseudomonas aeruginosa*, a bacterium that causes ventilator-associated pneumonia and lung infections in cystic fibrosis.

**Figure 5.**
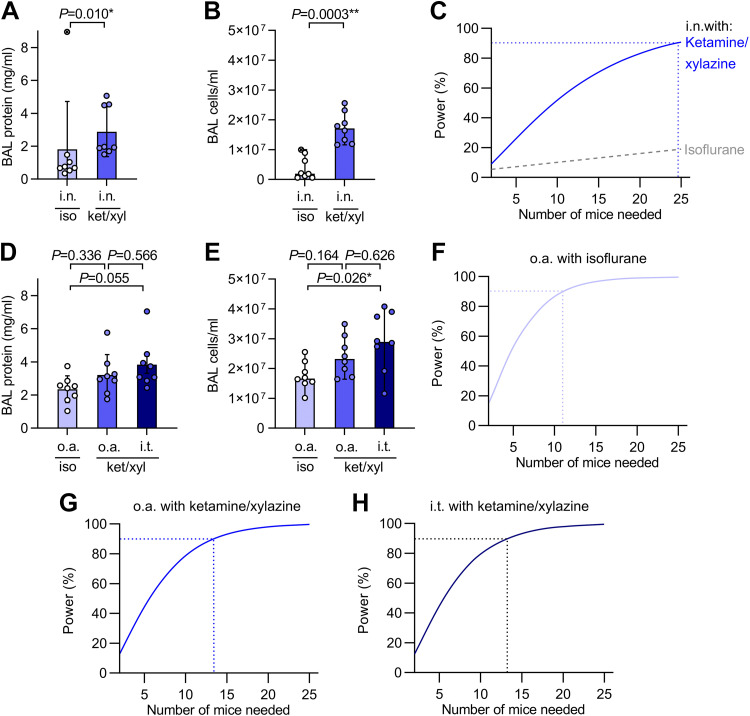
Anesthesia and administration approaches affect experimental power in a model of bacterial pneumonia. *A*. mice were randomized to receive either isoflurane (iso) or ketamine/xylazine (ket/xyl) anesthesia for intranasal (i.n.) dosing with 1×10^6^ colony forming units (c.f.u.) of *Pseudomonas aeruginosa* (PAO1). Bronchoalveolar lavage (BAL) supernatant protein concentration was measured at 24 h post infection. *B*: cell counts from BAL fluid at 24 h post infection. *C*: output of power analysis using BAL protein data in *A* to estimate number of mice needed per group to detect a 50% change in BAL protein concentration using an unpaired two-tailed *t* test. *D*: mice were randomized to receive 1×10^6^ c.f.u. of PAO1 either via oropharyngeal aspiration (o.a.) with isoflurane anesthesia, oropharyngeal aspiration with ketamine/xylazine anesthesia, or with intratracheal (i.t.) dosing under ketamine/xylazine anesthesia. BAL supernatant protein concentration was measured at 24 h post infection. *E*: cell counts from BAL fluid at 24 h post infection. *F*–*H*: output of power analyses using BAL protein data in *D* to estimate number of mice needed per group to detect a 50% change in BAL protein concentration using an unpaired two-tailed *t* test. Means ± standard deviation except for *B* and *E* which show medians ± 95% confidence intervals. Data from *B* and *E* were log_10_-transformed before analysis. *P* values are from Mann–Whitney *U* test (*A*); unpaired two-tailed *t* test (*B*); ordinary one-way ANOVA with Tukey’s multiple comparisons test (*D* and *E*), *n* = 8.

Similar to our data from the influenza A infection model, intranasal dosing with the PAO1 isolate of *P. aeruginosa* under isoflurane anesthesia resulted in less influx of protein and cells into the bronchoalveolar space than with ketamine/xylazine anesthesia (Fig. 5, *A* and *B*). One mouse in the isoflurane group developed a very high BAL protein response, driving increased variance and an even greater difference in estimated power between the two anesthesia approaches than we calculated in the viral infection model (Fig. 5*C*).

Relative to data with intranasal infection, delivery of the same bacterial inoculum via the oropharyngeal aspiration or intratracheal routes yielded greater increases in BAL protein and cell counts with lower variance (Fig. 5, *D* and *E*), giving increased experimental power (Fig. 5, *F*–*H*).

These results show how the improved pulmonary dosing efficiency was achieved with the oropharyngeal aspiration and intratracheal methods relative to intranasal dosing can reduce the number of mice needed for experiments.

## DISCUSSION

In this study, we found that anesthetic approach and administration route can affect the efficiency of fluid dosing to lungs of mice with impacts on experimental power in models of viral and bacterial pneumonia.

We conclude from our results that ketamine/xylazine anesthesia is preferable where consistent intranasal dosing to lungs of B6 mice is needed. As oropharyngeal aspiration dosing is simple, can be achieved under isoflurane anesthesia, and results in consistent pulmonary delivery, this route may be superior to intranasal dosing in some settings. Where precise dosing to lungs is critical, intratracheal dosing might be useful as the intratracheal administration approach that we describe resulted in high pulmonary dosing efficiency. Limitations of our intratracheal dosing approach include the requirement for intubation and the need for the use of a nose cone or additional ketamine sedation for use with isoflurane anesthesia.

A likely consequence of poor delivery to lungs using intranasal dosing under isoflurane anesthesia is that inoculating doses containing greater quantities of virus will be used to produce infections that consistently result in robust lung inflammation, exposing extrapulmonary tissues to higher quantities of virus. This is not desirable, as exposure of the nasal sinuses to high quantities of viral particles could cause serious adverse effects, as recently demonstrated in a study showing lethal SARS-CoV-2 neuroinvasion when K18-hACE2 mice were infected using intranasal dosing but not when mice were infected using aerosolization to more gradually initiate an infection with smaller droplets while producing a similar pulmonary viral load (24). Our results in the bacterial lung infection model indicate that oropharyngeal aspiration dosing might also be useful for improved modeling of viral pneumonia, and we speculate that retention and growth of bacteria in the nasal sinuses may also have contributed to the severe lung inflammation that developed in one of our mice given intranasal *P. aeruginosa* under isoflurane anesthesia.

Injectable ketamine/xylazine anesthesia may not always be preferable as recovery time can be longer than with isoflurane with potential effects on immune responses, and use of needles is discouraged where possible due to safety risks. In our study, we found that it was feasible to give mice intraperitoneal ketamine/xylazine injections in a biosafety cabinet separate from that used for handling virus to minimize infection risk to handlers. Limiting dose reflux using ketamine/xylazine anesthesia might also reduce the risk of aerosolization and surface contamination by inoculum, as well as animal suffering due to extrapulmonary pathology resulting from pathogen replication in the nasal sinuses (24).

Our study provides quantification of pulmonary dosing efficiency comparing intranasal, oropharyngeal aspiration, and intratracheal methods using anesthetics and mouse strains in current widespread usage. The percentage lung delivery values that we measured are largely consistent with those from previous studies that used a range of mouse strains, anesthetic approaches, and methods for measuring dose distribution (1, 4, 6, 14). Our conclusion that anesthetic type can alter lung deposition of intranasal doses differs from that of a previous study which found no effect of different anesthetics (isoflurane, halothane, and Avertin) during intranasal dosing on pulmonary dosing efficiency in BALB/c mice, although ketamine/xylazine was not examined in this previous report (1). Curiously, another study using BALB/c mice found increased bacterial content in lungs after intranasal dosing with *Francisella tularensis* under isoflurane compared with ketamine/xylazine anesthesia (25). B6 mice have wider bronchi than BALB/c mice (26), and *F. tularensis* given intranasally rapidly replicates outside of the lungs (27), so outcome of dosing may vary depending on mouse strain and pathogen biology.

In summary, we recommend the use of ketamine/xylazine anesthesia over isoflurane anesthesia for intranasal dosing into lungs of B6-background mice. Where needed, pulmonary dosing efficiency can be increased using the oropharyngeal aspiration route, and further still using intratracheal dosing. Anesthetic approach and administration method are factors that can alter outcomes of studies involving dosing to lungs, affecting the number of mice required for experiments. To increase reproducibility and decrease animal usage, these factors should be considered during experimental design and clearly reported in publications.

## DATA AVAILABILITY

Data will be made available upon reasonable request.

## GRANTS

We acknowledge support from NIH Grants R35HL161241 and R01AI160167 (to M. Looney); International Anesthesia Research Society Mentored Research Award (to C. Conrad); NIH T32 Research Training in Pediatric Critical Care Medicine 2T32HD049303-16 (to S. F. Moussavi-Harami); and American Society of Transplantation Research Network/CSL Behring Basic Research Grant, Association for the Advancement of Blood and Biotherapies Postdoctoral Grant, and Sandler Program for Breakthrough Biomedical Research Postdoctoral Independence Grant (to S. Cleary).

## DISCLOSURES

No conflicts of interest, financial or otherwise, are declared by the authors.

## AUTHOR CONTRIBUTIONS

C.C. and S.J.C. conceived and designed research; Y.S., L.Q., M.M., S.F.M.-H., and S.J.C. performed experiments; Y.S. and S.J.C. analyzed data; Y.S. and S.J.C. interpreted results of experiments; Y.S. and S.J.C. prepared figures; Y.S., M.R.L., and S.J.C. drafted manuscript; Y.S., M.M., C.C., S.F.M.-H., M.R.L., and S.J.C. edited and revised manuscript; Y.S., L.Q., M.M., C.C., S.F.M.-H., M.R.L., and S.J.C. approved final version of manuscript.

## References

[1] Southam DS, Dolovich M, O’Byrne PM, Inman MD. Distribution of intranasal instillations in mice: effects of volume, time, body position, and anesthesia. Am J Physiol Lung Cell Mol Physiol 282: L833–L839, 2002. doi:10.1152/ajplung.00173.2001. 11880310

[2] Meyer RE, Fish RE. A review of tribromoethanol anesthesia for production of genetically engineered mice and rats. Lab Anim (NY) 34: 47–52, 2005. doi:10.1038/laban1105-47. 16261153

[3] Gyorfi MJ, Kim PY. Halothane toxicity. In: StatPearls. Treasure Island, FL: StatPearls Publishing, 2023. 31424865

[4] Foster WM, Walters DM, Longphre M, Macri K, Miller LM. Methodology for the measurement of mucociliary function in the mouse by scintigraphy. J Appl Physiol (1985) 90: 1111–1117, 2001. doi:10.1152/JAPPL.2001.90.3.1111. 11181627

[5] MacDonald KD, Chang HYS, Mitzner W. An improved simple method of mouse lung intubation. J Appl Physiol (1985) 106: 984–987, 2009. doi:10.1152/JAPPLPHYSIOL.91376.2008. 19150857 PMC4587594

[6] Su X, Looney M, Robriquet L, Fang X, Matthay MA. Direct visual instillation as a method for efficient delivery of fluid into the distal airspaces of anesthetized mice. Exp Lung Res 30: 479–493, 2004. doi:10.1080/01902140490476382. 15524406

[7] Magnen M, Gueugnon F, Petit-Courty A, Baranek T, Sizaret D, Brewah YA, Humbles AA, Si-Tahar M, Courty Y. Tissue kallikrein regulates alveolar macrophage apoptosis early in influenza virus infection. Am J Physiol Lung Cell Mol Physiol 316: L1127–L1140, 2019. doi:10.1152/ajplung.00379.2018. 30908937

[8] Cleary SJ, Hobbs C, Amison RT, Arnold S, O’Shaughnessy BG, Lefrançais E, Mallavia B, Looney MR, Page CP, Pitchford SC. LPS-induced lung platelet recruitment occurs independently from neutrophils, PSGL-1, and P-selectin. Am J Respir Cell Mol Biol 61: 232–243, 2019. doi:10.1165/RCMB.2018-0182OC. 30768917 PMC6670039

[9] Collins FS, Tabak LA. NIH plans to enhance reproducibility. Nature 505: 612–613, 2014. doi:10.1038/505612A. 24482835 PMC4058759

[10] Freedman LP, Venugopalan G, Wisman R. Reproducibility2020: progress and priorities. F1000Res 6: 604, 2017. doi:10.12688/F1000RESEARCH.11334.1. 28620458 PMC5461896

[11] Matrosovich M, Matrosovich T, Garten W, Klenk HD. New low-viscosity overlay medium for viral plaque assays. Virol J 3: 63, 2006. doi:10.1186/1743-422X-3-63. 16945126 PMC1564390

[12] Ortiz-Muñoz G, Looney MR. Non-invasive intratracheal instillation in mice. Bio Protoc 5: e1504, 2015. doi:10.21769/BIOPROTOC.1504. 27390765 PMC4933024

[13] Watanabe A, Hashimoto Y, Ochiai E, Sato A, Kamei K. A simple method for confirming correct endotracheal intubation in mice. Lab Anim 43: 399–401, 2009. doi:10.1258/LA.2009.009008. 19535395

[14] Eyles JE, Spiers ID, Williamson ED, Alpar HO, Williamson ED. Tissue distribution of radioactivity following intranasal administration of radioactive microspheres. J Pharm Pharmacol 53: 601–607, 2001. doi:10.1211/0022357011775929. 11370699

[15] Galani IE, Triantafyllia V, Eleminiadou EE, Andreakos E. Protocol for influenza A virus infection of mice and viral load determination. STAR Protoc 3: 101151, 2022. doi:10.1016/J.XPRO.2022.101151. 35146450 PMC8819391

[16] D’Alessio FR. Mouse models of acute lung injury and ARDS. Methods Mol Biol 1809: 341–350, 2018. doi:10.1007/978-1-4939-8570-8_22. 29987799

[17] Pelgrim CE, van Ark I, Leusink-Muis T, Brans MAD, Braber S, Garssen J, van Helvoort A, Kraneveld AD, Folkerts G. Intratracheal administration of solutions in mice; development and validation of an optimized method with improved efficacy, reproducibility and accuracy. J Pharmacol Toxicol Methods 114: 107156, 2022. doi:10.1016/J.VASCN.2022.107156. 35085718

[18] Kunda NK, Price DN, Muttil P. Respiratory tract deposition and distribution pattern of microparticles in mice using different pulmonary delivery techniques. Vaccines (Basel) 6: 41, 2018. doi:10.3390/VACCINES6030041. 29996506 PMC6161314

[19] Barbayianni I, Ninou I, Tzouvelekis A, Aidinis V. Bleomycin revisited: a direct comparison of the intratracheal micro-spraying and the oropharyngeal aspiration routes of bleomycin administration in mice. Front Med (Lausanne) 5: 269, 2018. doi:10.3389/FMED.2018.00269. 30320115 PMC6165886

[20] Pantarelli C, Pan D, Chetwynd S, Stark AK, Hornigold K, Machin P, Crossland L, Cleary SJ, Baker MJ, Hampson E, Mandel A, Segonds-Pichon A, Walker R, van’t Veer C, Riffo-Vasquez Y, Okkenhaug K, Pitchford S, Welch HCE. The GPCR adaptor protein norbin suppresses the neutrophil-mediated immunity of mice to pneumococcal infection. Blood Adv 5: 3076–3091, 2021. doi:10.1182/BLOODADVANCES.2020002782. 34402884 PMC8405187

[21] Amison RT, O’Shaughnessy BG, Arnold S, Cleary SJ, Nandi M, Pitchford SC, Bragonzi A, Page CP. Platelet depletion impairs host defense to pulmonary infection with pseudomonas aeruginosa in mice. Am J Respir Cell Mol Biol 58: 331–340, 2018. doi:10.1165/rcmb.2017-0083OC. 28957635

[22] Ortiz-Muñoz G, Yu MA, Lefrançais E, Mallavia B, Valet C, Tian JJ, Ranucci S, Wang KM, Liu Z, Kwaan N, Dawson D, Kleinhenz ME, Khasawneh FT, Haggie PM, Verkman AS, Looney MR. Cystic fibrosis transmembrane conductance regulator dysfunction in platelets drives lung hyperinflammation. J Clin Invest 130: 2041–2053, 2020. doi:10.1172/JCI129635. 31961827 PMC7108932

[23] Conrad C, Yildiz D, Cleary SJ, Margraf A, Cook L, Schlomann U, Panaretou B, Bowser JL, Karmouty-Quintana H, Li J, Berg NK, Martin SC, Aljohmani A, Moussavi-Harami SF, Wang KM, Tian JJ, Magnen M, Valet C, Qiu L, Singer JP, Eltzschig HK, Bertrams W, Herold S, Suttorp N, Schmeck B, Ball ZT, Zarbock A, Looney MR, Bartsch JW; CAPSys Study Group. ADAM8 signaling drives neutrophil migration and ARDS severity. JCI Insight 7: e149870, 2022. doi:10.1172/JCI.INSIGHT.149870. 35132956 PMC8855804

[24] Fumagalli V, Ravà M, Marotta D, Di Lucia P, Laura C, Sala E, Grillo M, Bono E, Giustini L, Perucchini C, Mainetti M, Sessa A, Garcia-Manteiga JM, Donnici L, Manganaro L, Delbue S, Broccoli V, De Francesco R, D’Adamo P, Kuka M, Guidotti LG, Iannacone M. Administration of aerosolized SARS-CoV-2 to K18-hACE2 mice uncouples respiratory infection from fatal neuroinvasion. Sci Immunol 7: eabl9929, 2022. doi:10.1126/sciimmunol.abl9929. 34812647 PMC9835999

[25] Miller MA, Stabenow JM, Parvathareddy J, Wodowski AJ, Fabrizio TP, Bina XR, Zalduondo L, Bina JE. Visualization of murine intranasal dosing efficiency using luminescent francisella tularensis: effect of instillation volume and form of anesthesia. PLoS One 7: e31359, 2012. doi:10.1371/JOURNAL.PONE.0031359. 22384012 PMC3286442

[26] Thiesse J, Namati E, Sieren JC, Smith AR, Reinhardt JM, Hoffman EA, McLennan G. Lung structure phenotype variation in inbred mouse strains revealed through in vivo micro-CT imaging. J Appl Physiol (1985) 109: 1960–1968, 2010. doi:10.1152/JAPPLPHYSIOL.01322.2009. 20671036 PMC3006419

[27] Ojeda SS, Wang ZJ, Mares CA, Chang TA, Li Q, Morris EG, Jerabek PA, Teale JM. Rapid dissemination of *Francisella tularensis* and the effect of route of infection. BMC Microbiol 8: 215, 2008. doi:10.1186/1471-2180-8-215. 19068128 PMC2651876

